# A Comparison of the Preservation of Mouse Adipose Tissue-Derived Mesenchymal Stem Cells Using the University of Wisconsin Solution and Hank's Balanced Salt Solution

**DOI:** 10.1155/2018/1625464

**Published:** 2018-09-06

**Authors:** Saifun Nahar, Yoshiki Nakashima, Chika Miyagi-Shiohira, Takao Kinjo, Naoya Kobayashi, Issei Saitoh, Masami Watanabe, Hirofumi Noguchi, Jiro Fujita

**Affiliations:** ^1^Department of Infectious, Respiratory, and Digestive Medicine, Graduate School of Medicine, University of the Ryukyus, Okinawa 903-0215, Japan; ^2^Department of Regenerative Medicine, Graduate School of Medicine, University of the Ryukyus, Okinawa 903-0215, Japan; ^3^Department of Basic Laboratory Sciences, School of Health Sciences in the Faculty of Medicine, University of the Ryukyus, Okinawa 903-0215, Japan; ^4^Okayama Saidaiji Hospital, Okayama 704-8192, Japan; ^5^Division of Pediatric Dentistry, Graduate School of Medical and Dental Science, Niigata University, Niigata 951-8514, Japan; ^6^Department of Urology, Okayama University Graduate School of Medicine, Dentistry and Pharmaceutical Sciences, Okayama 700-8558, Japan

## Abstract

Preservation of adipose tissue before the isolation of cells is one of the most important steps in maintaining the cell viability of adipose tissue-derived mesenchymal stem cells (ADSCs) for clinical use. Hank's balanced salt solution (HBSS) is one of the main ADSC preservation solutions used clinically. However, this step is known to lead to decreased cell viability. The University of Wisconsin (UW) solution is recognized by transplant physicians as an excellent organ preservation solution. We aimed to investigate the effectiveness of UW solution in preservation of the viability of ADSCs. We collected adipose tissue from the inguinal fat pad of mice and compared preservation in UW solution and HBSS overnight by measuring cell viability after isolation. We found that the number of viable cells harvested per gram of adipose tissue mass was higher in UW solution- than HBSS-preserved tissue.

## 1. Introduction

Mesenchymal stem cells (MSCs) [1] are a source of cell therapy in regenerative medicine. MSCs have the ability to undergo self-renewal and to form differentiated cells, including adipocytes, chondrocytes, neuronal cells, and osteocytes [2]. MSCs also regulate biological functions such as growth factor release [3] and have immunosuppressive effects. Among several sources of MSCs, adipose tissue-derived mesenchymal stem cells (ADSCs) [4, 5] have become a renowned therapeutic tool in clinical use [6–8].

Most clinical researchers using ADSCs follow their own particular protocol for isolating ADSCs, and there is no optimal storage medium for preserving adipose tissue after collection. There are several reasons why facilities that perform cell therapy with ADSCs use a preserving solution. First, it can take from several hours to as long as a day to transport the tissue from the operating room to the cell processing center. Second, some clinical researchers believe that overnight preservation using a mixture of storage medium and penicillin/streptomycin is necessary to prevent bacterial infection. The selection of a storage solution is therefore critically important for maintaining the function of ADSCs for clinical use. Nevertheless, cell survival rates decrease when ADSCs are stored in preservation solution for 12–24 hours [9].

Hank's balanced salt solution (HBSS) [10, 11] and phosphate-buffered saline (PBS) solution are the most common solutions used for preserving certain types of cells. The essential function of HBSS is to maintain the pH and osmotic balance as well as provide the cells with water and inorganic ions. HBSS also contains glucose, which provides the cells with energy. Meanwhile, some studies have reported that the University of Wisconsin (UW) solution [12–14] has a better tissue preservation ability than other types of preservation solutions for certain types of cells [15].

Here, we compared the cell viability of ADSCs preserved in UW solution and HBSS.

## 2. Materials and Methods

### 2.1. Reagents

UW solution (Viaspan) was obtained from Astellas Pharma Inc. (Tokyo, Japan). HBSS and collagenase type IV solution were obtained from Thermo Fisher Scientific (Tokyo, Japan). All other materials used were of the highest commercial grade.

### 2.2. Animal Care

All experimental protocols were in accordance with the guidelines for the care and use of laboratory animals set by the Research Laboratory Center, Faculty of Medicine, and the Institute for Animal Experiments, Faculty of Medicine, University of the Ryukyus (Okinawa, Japan). The experimental protocol was approved by the Committee on Animal Experiments of the University of the Ryukyus (permit number: A2017101).

C57BL/6 male mice (8 weeks old; Japan SLC, Shizuoka, Japan) were maintained under controlled temperature (23 ± 2°C) and light conditions (lights on from 08:30–20:30). Animals were fed standard rodent chow pellets and had ad libitum access to water. All efforts were made to minimize the suffering of the animals.

### 2.3. Isolation of ADSCs from Adipose Tissue

**Figure 1:**
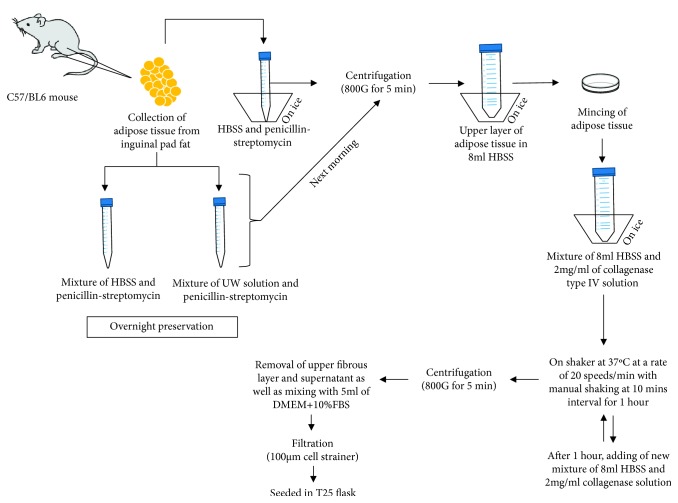
Illustration of the collection and preservation of adipose tissue-derived mesenchymal stem cells (ADSCs). ADSCs were isolated after storage of adipose tissue overnight in preservation solution (HBSS or UW solution). In the control group, ADSCs were isolated without preserving adipose tissue overnight.

Adipose tissue was extracted from the inguinal fat pad of 8-week-old mice (male, *n* = 3). The tissues were divided into three groups: nonpreserved control, HBSS-preserved, and UW solution-preserved groups. In the control group, ADSCs were isolated without preserving adipose tissue overnight. In the preservation groups, adipose tissue was submerged in HBSS or UW solution at 4°C for 16 h until the isolation procedure. Isolation of ADSCs from adipose tissue was conducted as previously described [16]. Briefly, the adipose tissue was stored in HBSS at 4°C temperature and washed vigorously three times using HBSS before tissue digestion. The adipose tissue was cut into small fragments using a scalpel and digested enzymatically (2 mg collagenase type IV/ml; HBSS) in 50 ml tubes with shaking (rotation speed 20 speeds/min at 37°C for 120 min; BioShaker BR-42FM, TAITEC, Saitama, Japan). The tubes were subsequently centrifuged (800 × g) for 5 minutes. The cell pellet containing ADSCs was collected and washed with fresh Dulbecco's Modified Eagle Medium (DMEM) containing 10% fetal bovine serum (FBS; Gibco-Invitrogen, Carlsbad, CA, USA) to remove residual enzyme after the digestion, and the digested cells were incubated in a T25 flask (illustration of the procedure is shown in Figure 1). All mouse studies were approved by the Institutional Animal Care and Use Committee of University of the Ryukyus.

### 2.4. Assessment of the Cellular Viability

The viability of ADSCs after isolation was assessed using 0.4% *w*/*v* Trypan blue solution (Wako Pure Chemical Corporation, Osaka, Japan) and C-Chip™ hemocytometers (NanoEnTek Inc., MA, USA).

### 2.5. Flow Cytometry

Cell flow cytometry was performed using a NovoCyte® Flow Cytometer (ACEA Biosciences Inc., San Diego, CA, USA) according to the manufacturer's instructions. Briefly, ADSCs (1 × 10^5^ cells) were mixed into 0.5 ml of Perfusion Solution (CORNING, Manassas, VA, USA). Each antibody (1/100 of the volume) was added to the cell mixture and incubated on ice for 30 minutes. After washing the cells with Brilliant Stain Buffer (BD Biosciences, Franklin Lakes, NJ, USA), fluorescence-activated cell sorting (FACS) measurements were conducted. The following primary antibodies were used: Brilliant Violet 421™ Rat Anti-Mouse CD44 (BD Biosciences), Fluorescein Isothiocyanate (FITC) Rat Anti-Mouse CD90.2 (BD Biosciences), PerCP/Cy5.5 Anti-Mouse CD34 (BioLegend, San Diego, CA, USA), and PE/Cy7 Rat Anti-Mouse CD45 (BD Biosciences). Isotype-identical antibodies were used as controls.

### 2.6. Real-Time PCR and RT-PCR

RNA was prepared for a qPCR using a RNeasy® Mini kit and QuantiTect® Reverse Transcription kit according to the manufacturer's instructions (Qiagen, Hilden, Germany). AmpliTaq Gold® 360 Master mix was used according to the manufacturer's instructions (Thermo Fisher Scientific, Tokyo, Japan). Real-time PCR analyses were performed using a LightCycler 96 Real-Time PCR system (Roche, Basel, Switzerland). The Taqman® Universal Master Mix II, no UNG (Applied Biosystems, USA) was used according to the manufacturer's instructions. An RT-PCR was performed using a GeneAtlas 482 thermal cycler (Astec Co. Ltd., Fukuoka, Japan). Images were recorded using an Aplegen® Omega Lum C (Gel Company, San Francisco, CA, USA).

The primers used for the RT-PCR included the following:

- Mouse HGF forward, ACTCTTACCAAGGAAGACCCATTAC
- Mouse HGF reverse, ATACCAGTAGCATCGTTTTCTTGAC
- Mouse EGF forward, TATCCATCGGTAATAAGGGTGAAC
- Mouse EGF reverse, CCAGCACACACTCATCTATATCAGA
- Mouse VEGF forward, TGTCTTCACTGGATATGTTTGACTG
- Mouse VEGF reverse, TTCTCTGTCATCATCTGTCTCTCTG
- Mouse SCAI forward, GGTTGTTTTCAGTACCTCTTTCCTG
- Mouse SCAI reverse, CATGCTCATTACTAGAAAAGCCAGG
- Mouse GAPDH forward, AACTCACTCAAGATTGTCAGCAATG
- Mouse GAPDH reverse, GCTGTAGCCGTATTCATTGTCATAC

### 2.7. Cell Proliferation

The cells were seeded onto 24-well plates at a density of 1.0 × 10^4^ cells/well. The slides were photographed with a microscope (×40) and the number of cells in the image was counted. The relative cell proliferation rate was calculated by converting the number of cells on the day of cell seeding to 100%.

### 2.8. Cell Differentiation

Adipogenic differentiation was examined using OriCell™ Mesenchymal Stem Cell Adipogenic Differentiation Medium (GUXMX-90031, Cyagen Biosciences) and the Lipid Assay Kit (AK09F, Cosmo Bio Co. Ltd, Tokyo, Japan) according to the manufacturer's instructions. Osteogenic differentiation was examined using OriCell™ Mesenchymal Stem Cell Osteogenic Differentiation Medium (GUXMX-90021, Cyagen Biosciences) and the Calcified Nodule Staining Kit (AK21, Cosmo Bio Co. Ltd, Tokyo, Japan) according to the manufacturer's instructions.

### 2.9. Statistical Analyses

The data are presented as the mean ± standard error (SE). The differences among three groups were analyzed using two-way ANOVA. *P* values of <0.05 were considered statistically significant.

## 3. Results and Discussion

### 3.1. Characteristics of ADSCs Isolated without Preservation

**Figure 2:**
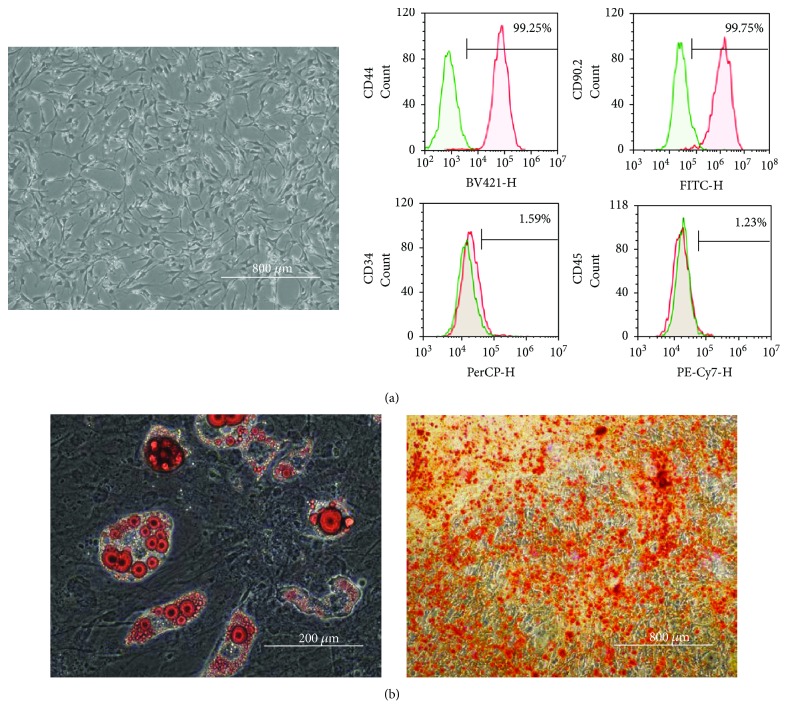
Phenotype and differentiation potential of ADSCs isolated without preservation, which served as control group. Morphological appearance of ADSCs (passage 2) (a, left panel) and cell surface markers of ADSCs by flow cytometry (passage 2) (a, right panel). Representative images of adipocyte (b, left panel) and osteocyte differentiation (b, right panel) from mouse ADSCs (passage 2) cultured in differentiation medium.

ADSCs isolated without preservation served as the control group. They were cultured to 80% confluence in DMEM containing 10% FBS. Microscopy was performed to confirm the absence of abnormalities with regard to cell size and shape and culture state (Figure 2(a)). Flow cytometry was performed using markers of mouse MSCs (CD44 and CD90.2), hematopoietic stem cells (CD34), and leukocytes (CD45). We observed the expression of CD44 and CD90.2 but not CD34 and CD45 (Figure 2(a)), confirming that the isolated cells were MSCs. We induced differentiation of the ADSCs into adipocytes (Figure 2(b)) and osteoblasts (Figure 2(b)). Mature adipocytes were stained with Oil Red O, and mature osteoblasts were stained with Alizarin Red S.

### 3.2. Cell Viability of ADSCs after Preservation in HBSS or UW Solution

**Figure 3:**
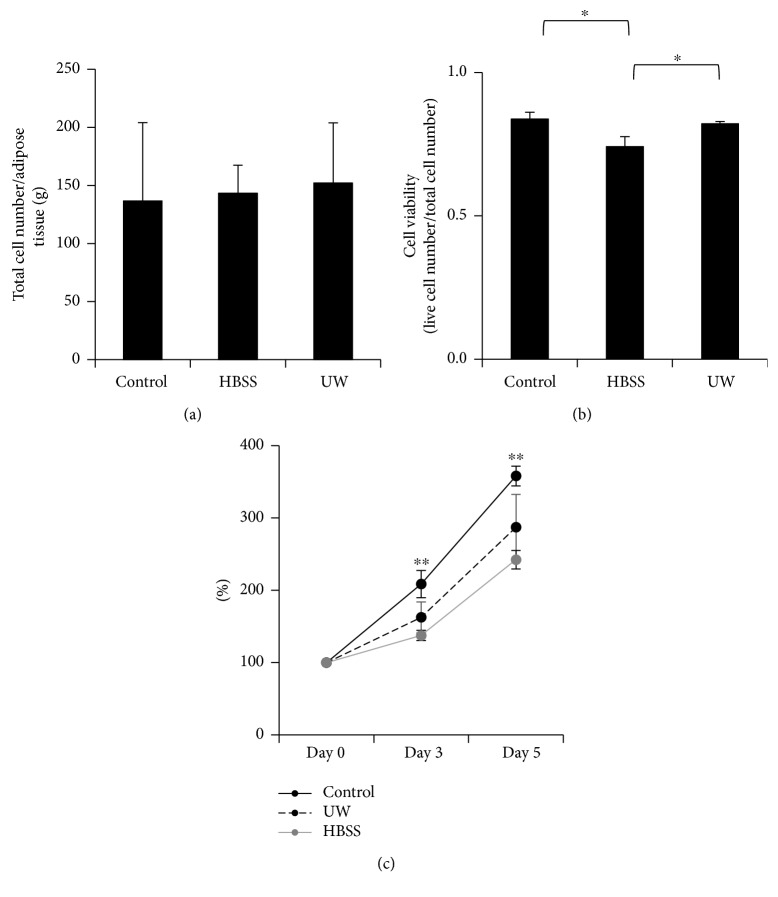
Total cell number and cell viability of ADSCs after preservation for 16 h in Hank's balanced salt solution (HBSS) or University of Wisconsin (UW) solution. Graphical representation of the total number of cells yielded from 1 gram of mouse adipose tissue after overnight preservation in UW and HBSS. ADSCs isolated without preservation served as control group (a). Cellular viability was determined by counting the number of live cells (not stained with 0.4% *w*/*v* Trypan blue solution) under a microscope. The decrease in cell viability of the HBSS group compared with both the control and UW groups was significant. Data are expressed as the mean ± SE (control group: 0.83 ± 0.03, HBSS group: 0.74 ± 0.03; ^∗^ *P* < 0.05. UW solution group: 0.82 ± 0.01; ^∗^ *P* < 0.05, *n* = 3) (b). Cell proliferation rate in the HBSS group on days 3 and 5 was significantly lower than that of the control group. Data are mean ± SE. The relative value is shown with the number of cells on day 0 as 100%. Day 0 (control group: 100.0 ± 0.0, UW group: 100.0 ± 0.0, HBSS group: 100.0 ± 0.0, *n* = 5). Day 3 (control group: 208.5 ± 18.9, UW group: 162.5 ± 21.2, HBSS group: 137.5 ± 7.1; ^∗∗^ *P* < 0.01, *n* = 5). Day 5 (control group: 358.0 ± 13.7, UW group: 287.0 ± 45.4, HBSS group: 242.3 ± 12.9; ^∗∗^ *P* < 0.01, *n* = 5) (c).

To assess the cell viability in each group, we measured the total cell number/tissue weight (mg) and live cell number/tissue weight (mg). There were no significant differences in the total cell number between the control group (1.37 ± 0.43 × 10^7^/g) and two preservation groups (HBSS group, 1.43 ± 0.16 × 10^7^/g; UW solution group, 1.52 ± 0.33 × 10^7^/g) (*n* = 3) (Figure 3(a)). These data suggest that the isolation of ADSCs in the control and two preservation groups were of similar processing efficiency. We also calculated the live cell number/total cell number ratio. There was a significant difference in cell viability between the control and HBSS-preserved group but not the UW-preserved group (control group: 0.83 ± 0.03, HBSS group: 0.74 ± 0.03, UW solution group: 0.82 ± 0.01) (*n* = 3) (Figure 3(b)). These data suggest that the cell viability of ADSCs after preservation in HBSS was significantly decreased compared to both the control and UW solution-preserved groups. ADSCs in each group were seeded at a concentration of 1 × 10^4^ cells/24 well and the number of cells on days 3 and 5 was then counted (Figure 3(c)). The cell proliferation rate on days 3 and 5 of the UW group was equivalent to that of the control group. However, the cell proliferation rate on days 3 and 5 of the HBSS group was significantly lower than that of the control group. ADSC preserved in UW solution maintained a cell proliferation ability equivalent to that of the control group.

### 3.3. Characteristics of the Isolated ADSCs after Preservation Using HBSS or UW Solution

**Figure 4:**
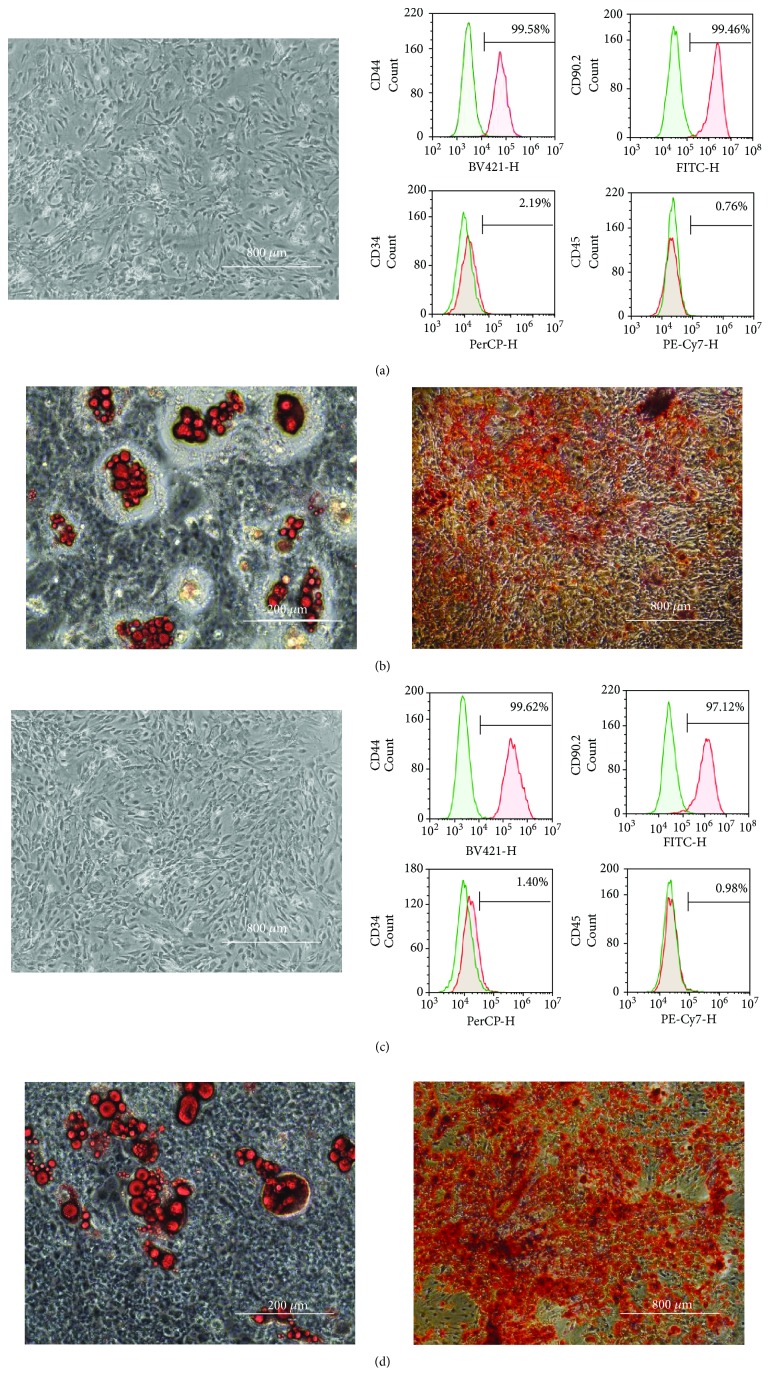
Phenotype and differentiation potential of ADSCs after preservation for 16 h in HBSS or UW solution. Morphological appearance of ADSCs (passage 2) in the HBSS- (a, left panel) and UW solution-preserved groups (c, left panel). Cell surface markers of ADSCs (passage 2) preserved in HBSS (a, right panels) or UW solution (c, right panels) by flow cytometry. Representative images of differentiated adipocytes from ADSCs preserved in HBSS (b, left panel) and UW solution (d, left panel), and differentiated osteocytes from ADSCs preserved in HBSS (b, right panel) and UW solution (d, right panel).

The characteristics of mouse ADSCs after preservation are shown in Figure 4. ADSCs were cultured to 80% confluence in DMEM medium containing 10% FBS. Microscopy was performed to confirm the absence of abnormalities with regard to cell size and shape and the culture state in the HBSS- (Figure 4(a)) and UW solution-preserved groups (Figure 4(c)). Flow cytometry was performed using markers of mouse MSCs (CD44, CD90.2), hematopoietic stem cells (CD34), and leukocytes (CD45). We observed the expression of CD44 and CD90.2 but not CD34 or CD45 in cells that were preserved in HBSS (Figure 4(a)) and UW solution (Figure 4(c)), indicating the presence of MSCs. We confirmed the expression level of ADSC surface markers (CD34, CD44, and CD45) of the control, HBSS and UW groups using the PCR measurement method. Results were similar to those of flow cytometer analysis (data not shown). PCR was used to examine the mRNA expression levels of hepatocyte growth factor (HGF), epidermal growth factor (EGF), vascular endothelial growth factor (VEGFA), and a suppressor of cancer cell invasion (SCAI) expressed in ADSC. As a result, cell functions of the control, HBSS and UW groups were evaluated as equivalent (Supplemental Figure 1A). We induced differentiation of ADSCs in the HBSS (Figure 4(b)) and UW solution-preserved groups (Figure 4(d)) into adipocytes. We also induced differentiation of the ADSCs in the HBSS (Figure 4(b)) and UW solution-preserved groups (Figure 4(d)) into osteoblasts. The presence of mature adipocytes and osteoblasts was confirmed by staining with Oil Red O and Alizarin Red S, respectively. Maintenance of differentiation potential was demonstrated as osteogenic and adipogenic differentiation.

Adipose tissue is considered a reliable and abundant source of adult stem cells for regenerative therapy. To yield good quality ADSCs, adipose tissue must be preserved in an appropriate solution before isolation. We investigated the effectiveness of two storage solutions (HBSS and UW solution) for preserving adipose tissue overnight before isolation of ADSCs by examining subsequent cell viability. We found that cell viability was higher in the UW solution group (82%) than the HBSS group (74%). However, UW solution has an inhibitory effect on collagenase digestion. That tissue digestion is the most important step in the isolation of ADSCs from adipose tissue makes this a major disadvantage. To overcome this, we washed the adipose tissue with HBSS or Dulbecco's phosphate-buffered saline (DPBS).  Table 1 shows the compositions of UW solution and HBSS. The UW solution contains higher potassium (120 mmol/l), magnesium (5 mmol/l), and phosphorus (25 mmol/l) than HBSS. It also contains many additional components such as lactobionate (100 mmol/l), raffinose (30 mmol/l), adenosine (5 mmol/l), allopurinol (1 mmol/l), and glutathione (3 mmol/l). These components might have contributed to maintaining the survival rate of ADSCs. However, the UW solution is ten times more expensive than the HBSS solution. Meanwhile, the composition of HBSS is similar to that of PBS and is characterized by its containing glucose (5.6 mmol/l) and low price. Interestingly, however, our results suggest that there is almost no influence of glucose on adipose tissue preservation for 16 h. This is in agreement with previous reports that revealed that glucose concentration does not affect the cell proliferation of human MSCs [17]. In other reports, however, bone marrow-derived MSCs were observed to have fewer mitochondria in the glycolysis system and lower glucose metabolism [18]. In addition, there are reports that high glucose promotes bone differentiation of MSCs [19]. Therefore, glucose containing preservation solution may not be necessary to maintain quality of ADSCs.

## 4. Conclusions

UW solution has been confirmed to significantly enhance cell survival and cell proliferation ability as a preservation solution of ADSCs. However, considering that UW solution is substantially more expensive than HBSS, our findings also suggest that HBSS possess sufficient function as a preservative solution.

## Figures and Tables

**Table 1:** The specific composition of UW solution and HBSS.

	University of Wisconsin (UW) solution	Hank's balanced salt solution (HBSS)
Natrium [Na^+^] (mmol/l)	30	142.8
Kalium [K^+^] (mmol/l)	120	5.8
Magnesium [Mg^2+^] (mmol/l)	5	0.9
Calcium [Ca^2+^] (mmol/l)	—	1.3
Carbonate [CO3^2−^] (mmol/l)	—	4.2
Chlorine [Cl^−^] (mmol/l)	—	146.8
Phosphate [PO4^3−^] (mmol/l)	25	0.8
Sulfate [SO4^2−^] (mmol/l)	5	0.4
Lactobionate (mmol/l)	100	—
Raffinose (mmol/l)	30	—
Adenosine (mmol/l)	5	—
Allopurinol (mmol/l)	1	—
Glutathione (mmol/l)	3	—
Glucose (mmol/l)	—	5.6
HES (g/l)	50	—
pH	7.4	7.2

## Data Availability

The data used to support the findings of this study are available from the corresponding author upon request.

## Supplementary Materials

Supplementary Materials Supplemental Figure 1: assay of growth factor expression level of ADSC after storage in HBSS or UW solution for 16 hours. Results of RT-PCR to evaluate growth factor and cell surface marker mRNA expression of ADSCs (Supplemental Figure 1A). Full-length images of the blots used for Supplemental Figure 1A (Supplemental Figure 1B).
