# Supplementary figures and images for: A Comparison of the Preservation of Mouse Adipose Tissue-Derived Mesenchymal Stem Cells Using the University of Wisconsin Solution and Hank's Balanced Salt Solution
Source: Stem Cells Int. 2018 Sep 6;2018:1625464. doi: 10.1155/2018/1625464 (PMC6146634; doi:10.1155/2018/1625464)

**A**

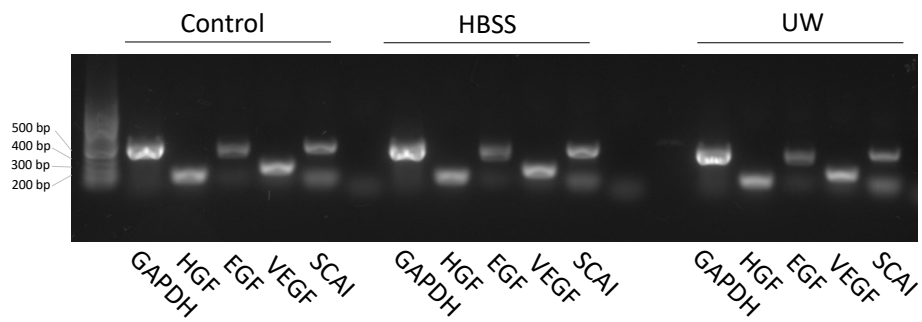

**B**

Full-length images of the blots  
used for Sup Fig. 1A

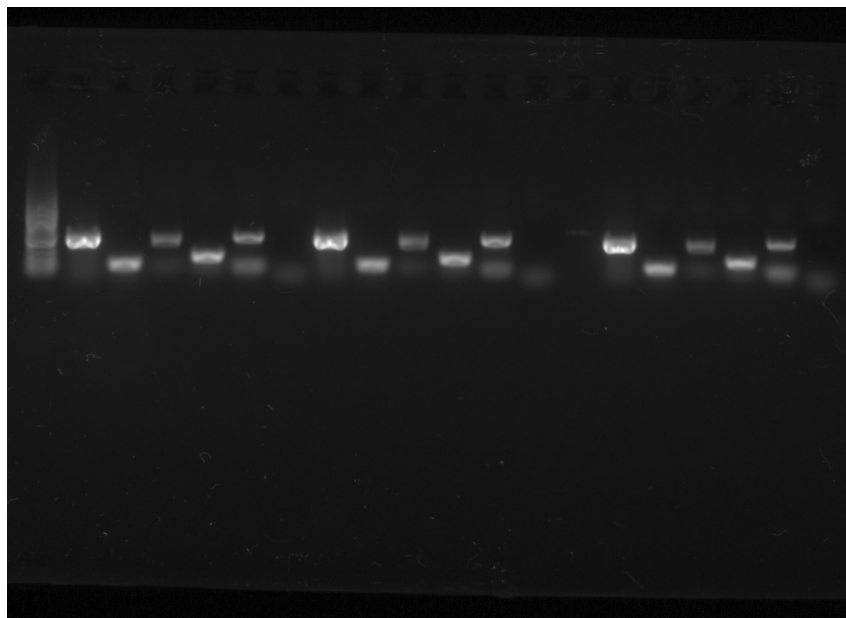

Supplement: Supplementary Materials — Supplemental Figure 1: assay of growth factor expression level of ADSC after storage in HBSS or UW solution for 16 hours. Results of RT-PCR to evaluate growth factor and cell surface marker mRNA expression of ADSCs (Supplemental Figure 1A). Full-length images of the blots used for Supplemental Figure 1A (Supplemental Figure 1B). [file 1625464.f1.pdf]
